# Prognostic-related genes for pancreatic cancer typing and immunotherapy response prediction based on single-cell sequencing data and bulk sequencing data

**DOI:** 10.32604/or.2023.029458

**Published:** 2023-07-21

**Authors:** XUEFENG WANG, SICONG JIANG, XINHONG ZHOU, XIAOFENG WANG, LAN LI, JIANJUN TANG

**Affiliations:** 1 Department of Respiratory and Critical Care Medicine, The First Affiliated Hospital of Nanchang University, National Regional Center for Respiratory Medicine, Nanchang, 330006, China; 2 Division of Thoracic and Endocrine Surgery, University Hospitals and University of Geneva, Geneva, Switzerland; 3 Department of Hepatobiliary Surgery, Xiantao First People’s Hospital of Yangtze University, Xiantao, 433099, China; 4 Department of Gastrointestinal Surgery, The First Affiliated Hospital, Sun Yat-sen University, Guangzhou, 510062, China; 5 Department of Radiation Oncology, The Third Affiliated Hospital of Kunming Medical University/Yunnan Cancer Hospital, Kunming, 650118, China

**Keywords:** Pancreatic cancer, Molecular subtypes, Single-cell sequencing, Immune microenvironment, Tumor immunity

## Abstract

**Background:**

Pancreatic cancer is associated with high mortality and is one of the most aggressive of malignancies, but studies have not fully evaluated its molecular subtypes, prognosis and response to immunotherapy of different subtypes. The purpose of this study was to explore the molecular subtypes and the key genes associated with the prognosis of pancreas cancer patients and study the clinical phenotype, prognosis and response to immunotherapy using single-cell seq data and bulk RNA seq data, and data retrieved from GEO and TCGA databases.

**Methods:**

Single-cell seq data and bioinformatics methods were used in this study. Pancreatic cancer data were retrieved from GEO and TCGA databases, the molecular subtypes of pancreatic cancer were determined using the six cGAS-STING related pathways, and the clinical phenotype, mutation, immunological characteristics and pathways related to pancreatic cancer were evaluated.

**Results:**

Pancreatic cancer was classified into 3 molecular subtypes, and survival analysis revealed that patients in Cluster3 (C3) had the worst prognosis, whereas Cluster1 (C1) had the best prognosis. The clinical phenotype and gene mutation were statistically different among the three molecular subtypes. Analysis of immunotherapy response revealed that most immune checkpoint genes were differentially expressed in the three subtypes. A lower risk of immune escape was observed in Cluster1 (C1), indicating higher sensitivity to immunotherapeutic drugs and subjects in this Cluster are more likely to benefit from immunotherapy. The pathways related to pancreatic cancer were differentially enriched among the three subtypes. Five genes, namely SFRP1, GIPR, EMP1, COL17A and CXCL11 were selected to construct a prognostic signature.

**Conclusions:**

Single-cell seq data were to classify pancreatic cancer into three molecular subtypes based on differences in clinical phenotype, mutation, immune characteristics and differentially enriched pathways. Five prognosis-related genes were identified for prediction of survival of pancreatic cancer patients and to evaluate the efficacy of immunotherapy in various subtypes.

## Introduction

Pancreatic cancer is associated with high mortality, ranking third in cancer-related deaths globally. The 5-year survival rate of pancreatic cancer is 3%. The incidence of pancreatic cancer is 1.6%, but the incidence increases due to smoking, obesity and other related factors [1]. Exocrine pancreatic ductal adenocarcinoma (PDAC) represents 90% of all the pancreatic cancer cases, whereas endocrine pancreatic carcinoma accounts for less than 5% of all the cases [2]. Diagnosis of pancreatic cancer at an early stage is challenging due to the lack of specific clinical symptoms and effective diagnostic methods. This cancer is characterized by high aggressiveness, but most cases are diagnosed in the late stage, and only 15% and 20% of the patients are eligible for surgery at diagnosis [3].

Surgery, chemotherapy, radiotherapy and palliative treatment are the conventional treatment strategies for the pancreatic cancer. Surgery is the curative treatment option for pancreatic cancer [4]. Neoadjuvant chemoradiotherapy effectively shrinks the tumor, thus some patients with advanced disease can undergo surgery and achieve longer expected survival [5]. Nevertheless, these subjects can develop resistance to chemotherapy due to the metastatic and aggressive nature of pancreatic cancer [6]. Therefore, this treatment option may not be effective in most patients. Immunotherapy has become an important treatment strategy in addition to surgery and chemotherapy [7], however, its efficacy against pancreatic cancer is limited compared with other tumors due to resistance induced by innate or adaptive immune effects [8,9]. As a result, various treatment plans and individualized therapy have been proposed to improve the outcomes of pancreatic cancer patients.

Cancers were previously classified according to their tissue of origin and clinical decisions on different treatment plans were made based on this classification. However, subsequent studies reported that patients with pathologically identical tumors had different prognoses. and Subtyping of cancers based on the similarities and differences in biologically relevant molecular similarities and differences can improve the conventional classification methods. Conventional classification methods can be optimized through accurate morphological and imaging analysis to improve the selection of systemic treatment regimens, individualized patient management, (primary surgery and neoadjuvant therapy), recruitment to clinical trials based on response prediction, and the evolution of treatment and research [10]. A recent study reported differences in gene expression within cells and altered interactions between cancer cells and various components of the tumor microenvironment (TME) [11]. Several cancer treatment options are being optimized or emerging due to advances in molecular typing. However, these methods have not been fully developed for pancreatic cancer compared with other cancers, such as breast and colon cancer. Moreover, the clinically relevant morphological or molecular subtypes of pancreatic cancer have not been fully elucidated [10]. A combination of immune gene expression and tumor cell expression analysis can provide essential information for developing methods for pancreatic cancer treatment [12].

Chronic pancreatitis is a risk factor for pancreatic cancer, and previous studies report a 7.2-fold higher risk for patients with a history of pancreatitis [13–17]. The sustained activation of the cGAS-STING pathway and its downstream effectors are associated with chronic inflammation and progression of cancer [18,19]. Therefore, the role of the cGAS-STING pathway in pancreatic cancer worth an in-depth study.

Single-cell sequencing is the amplification and sequencing of RNA or DNA extracted from a single cell. This technique can be used to accurately determine the genetic profile and expression status of a single cell, providing a basis for evaluating the heterogeneity of cells with the same phenotype [20]. In addition, this technique can provide new clues and insights into the mechanisms of tumorigenesis, metastasis and progression, the origin of tumors, differentiation of tumor stem cells and resistance to therapy. Therefore, the technique has high theoretical potential in evaluating the molecular subtypes of pancreatic cancer.

TME is the cellular environment around tumors, including tumor cells, fibroblasts, mesenchymal cells, blood and lymphatic vessels, as well as various tumor-infiltrating immune cells and related chemokines and cytokines [21]. TME plays a critical role in tumorigenesis and progression [22,23]. Exploring the various biological processes that occur in TME and tumor immune microenvironment (TIME) is essential for exploring tumor evolution mechanism and developing new tumor immunotherapy options [24]_._

In this study, we comprehensively evaluated the association between the molecular subtypes of pancreatic cancer and immune microenvironment. In addition, the correlation between expression of key genes and tumor immunity was investigated. The roles of critical genes in pancreatic cancer molecular subtypes were explored. These findings provide insight into underlying prognostic biomarkers related to immune infiltration of pancreatic cancer. Further, a comparison of the pathological features of the various molecular subtypes of pancreatic cancer was conducted and a risk model was constructed using the identified key genes.

## Methods

### scRNA data retrieval

Single-cell sequencing data (GSE154778 dataset) were retrieved from NCBI Data GEO (http://www.ncbi.nlm.nih.gov/geo/), and grouped into three pancreatic cancer datasets.

### TCGA data retrieval and pre-processing

Data on clinical phenotypes of pancreatic cancer were retrieved from the TCGA database (https://portal.gdc.cancer.gov/), comprising 176 tumor samples.

CNV mutation data on the Masked Copy Number Segment type of pancreatic cancer were obtained from TCGA database. The CNV results were integrated using Gistic2 software.

Mutect2 software was used to calculate the SNV mutation information of the TCGA-PAAD gene.

### GEO data retrieval and pre-processing

Five sets of microarray data were retrieved from GEO database. The data comprised 42 tumor tissues from the GSE28735 dataset, 63 tumor tissues from the GSE57495 dataset, 66 tumor tissues from the GSE62452 dataset, 125 tumor tissues from the GSE71729 dataset, and 79 tumor tissues from the GSE85916 dataset.

The “limma” and “sva” packages in R were used to eliminate the batches of each sample and the “normalizeBetweenArrays” function were used for standardization of the five datasets.

### cGAS-sting-related pathway retrieval

The six pathways associated with cGAS-STING were obtained based previous literature [25].

### Single-cell cluster dimension reduction analysis

The single cell data was screened and 10,232 cells were obtained. The percentage of rRNA and mitochondria was calculated using the “PercentageFeatureSet” function in R and 9,292 cells were obtained.

The “log-normalization” function in R was used to normalize the data of the three pancreatic cancer samples downloaded from the GEO database. The highly variable genes were identified using “FindVariableFeatures” function in R. Batch effects were eliminated using the CCA method in “FindIntegrationAnchors” and “IntegrateData” functions. A dim = 35 was chosen and the cells were Clustered using the “FindClusters” and “FindNeighbors” functions (with resolution = 0.1) by scaling all genes using the “ScaleDate” function and the anchors were identified through “PCA” dimensionality reduction.

Immune cells were extracted and re-Clustered by Resolution = 0.1, resulting in 10 subpopulations. UMAP (Uniform Manifold Approximation and Projection) dimensionality reduction analysis was performed on 9884 cells using the “RunTSNE” function, annotating with classical marker.

Marker genes of the six subgroups were screened using the “FindAllMarkers” function with a logFC of 0.5 (difference ploidy), a minpct (minimum percentage of differentially expressed genes) of 0.5 and an adjusted *p* < 0.05.

Further, the proportions of these six subgroups in each sample were determined BP enrichment analysis was performed using the “ClusterProfiler” package.

### Dysregulation of immune cells TME

The “copykat” package was used to predict “CNV” changes in scRNA-seq immune cell data to distinguish tumor cells from normal cells in each sample. cGAS.STING related pathways were retrieved and the scores of malignant and non-malignant cells were calculated using “ssGSEA” method in “GSVA” package. The “z-score” was standardized based on the enrichment score for each pathway.

### Identification of key genes in Bulk RNA-seq

ssGSEA was performed to determine the score of cGAS.STING-related pathways and the CD8 T score of patients in the TCGA dataset. The genes encoding the proteins were correlated with the cGAS.STING-related pathway score and CD8 T score by pearson correlation analysis, respectively. The key genes were identified using the criteria: |cor| > 0.5, *p* < 0.001.

Prognostic genes were identified from the key genes through univariate Cox analysis using the survival function in R (*p* < 0.01).

### Identification of pancreatic cancer molecular subtypes based on CD8 T cells and genes associated with cGAS.STING pathways

The “Pearson” and “PAM” algorithms were employed as the metrics distance and 500 bootstraps were performed to identify the molecular subtypes using the “ConsensusClusterPlus” R package. The training set comprised 80% of the patients. Tumor tissue in the TCGA dataset were used to classify patients based on consistent Clustering of 26 key gene expression profiles. The optimal number of Clusters was determined based on the cumulative distribution function (CDF).

The “limma” and “sva” packages were used to eliminate batch effects from the five GEO datasets. The “normalizeBetweenArrays” function was used to re standardize the data.

### Comparison of the clinical phenotypes of molecular subtypes

The distribution of different clinical features in the TCGA dataset were compared among the three molecular subtypes to assess whether the clinical features differed among the subtypes (chi-square test).

### Mutational characteristics of the molecular subtypes

The SNV mutation data were obtained from the TCGA dataset using mutect2. The first ten genes with the most significant mutations were selected from every subtype. The distributions of Fraction Altered, Homologous Recombination Defects, tumor mutation burden and Number of Segments were compared among the subtypes.

### Immunologic characteristics of molecular subtypes and differences between immunotherapy and chemotherapy

The level of infiltration of immune cells in the TCGA cohort were evaluated to determine the differences in the immune microenvironment of patients among various molecular subtypes based on the levels of gene expression in immune cells. The scores of the 22 immune cells were evaluated using the “CIBERSORT” algorithm and “Kruskal.test” was conducted to identify differences between the three subtypes. “ESTIMATE” tool was utilized to evaluate the level of immune cell infiltration. The expression levels of immune checkpoint genes in the three subtypes were evaluated.

We then evaluated the difference in immunotherapy efficacy between the different subtypes. The clinical effectiveness of immunotherapy in the molecular subtypes was evaluated through using the “TIDE” (http://TIDE.dfci.harvard.edu/) tool.

### Pathway analysis of molecular subtypes

The pathway scores for the various molecular subtypes of the samples were calculated using the “c2.cp.kegg.v7.5.1.symbols.gmt” function in the “GSVA” package.

GSEA was conducted using the GSEA software with “h. all. v7.5. symbols. gmt” as the background set.

The differences in 10 oncogenic pathways reported in previous studies [26] were evaluated in three molecular subtypes.

### Construction of risk models on the basis of key phenotypic genes of CD8 T and cGAS.STING cells

Differential analysis for Clust1 *vs*. no_Clust1, Clust2 *vs*. no_Clust2, and Clust3 *vs*. no_Clust3 was conducted using “Limma” package in R. FDR < 0.05 and |log2(Fold Change)| > 1 were used as the criteria.

A single-factor Cox regression analysis was conducted using ‘survival’ package to identify differentially expressed genes with *p* < 0.01.

We used lasso (Least absolute shrinkage and selection operator, Tibshirani (1996)) regression to further compress this prognosis-related genes in the TCGA dataset to reduce the number of genes in the risk model. Further we used stepwise multifactor regression analysis based on the genes from the lasso analysis results, starting with the most complex model using the stepAIC method in the “MASS” package and sequentially removing one variable to reduce the AIC (Akaike information criterion).

The TCGA dataset was utilized as the training dataset and risk scores were calculated separately for each sample based on the expression levels of prognosis-related genes. ROC analysis of the prognostic classification of the Riskscore was carried out with the “timeROC” R package. The prognostic classification efficiency was evaluated at 1, 3 and 5 years. At the same time, the zscore of Riskscore was conducted, and samples with Riskscore greater than zero were classified as high-risk group and samples with less than zero were classified as low-risk group after zscore, and KM survival curves were generated. To better validate the robustness of the model, the merged GSE dataset is validated using the same method.

### Evaluation of the Riskscore using samples with different clinicopathological features

The relationship between clinical tumor features and Riskscore scores between the different clinical phenotypes of the TCGA dataset were evaluated to determine its clinical significance.

### Integration of Riskscore with clinicopathological characteristics to improve prognostic models and survival prediction

The risk scores were combined with clinical features and with univariate and multivariate Cox regression analyses conducted. A nomogram was constructed by combining the Riskscore with other clinicopathological characteristics for the risk assessment and survival prediction.

### Validation of the expression levels of the selected genes through qRT-PCR

Chondrocytes from normal human (C-7, Procell, Wuhan, Hubei, China) and pancreatic cancer patients (panc-1, patu8988, bxpc-3 Hoge Biotechnology Co., Ltd., Shanghai, China) were cultured in chondrocytes containing 10% fetal bovine serum (FBS, 10099, Thermo Fisher Scientific, Massachusetts, USA) in DMEM/F12 medium (SH30126.01, HyClone Technologies, Logan, USA). The relative expression of EMP1, GIPR, SFRP1, CXCL11, and COL17A mRNA was detected after 48 h of incubation. Primers were designed using DNAMAN software and synthesized by Shanghai Biotechnology Co., China. Cellular RNA was extracted using TRIzol (Invitrogen #15596-026). cDNA was synthesized using PrimeScript™ RT kit with gDNA Eraser (Takara #RR047A) and SYBR Green qPCR Mix (Beyotime #D7260). Amplification cycle was 40 cycles using 7500 Real-Time Polymerase Chain Reaction (RT-PCR) system. PCR data were treated with GAPDH as an internal reference and the relative expression in the samples was calculated using the ΔΔCT method.

## Results

### Single-cell clustering and dimensionality reduction analysis

The results revealed that the amounts of mRNA and UMI were significantly correlated, but the amount of UMI/mRNA was not significantly correlated with the expression level of mitochondrial genes (Suppl. Fig. S1A). A violin plot before and after quality control of the data is presented in Suppl. Figs. S1B and S1C.

Suppl. Fig. S1D is the sample distribution graph and the anchor point graph of PCA. Finally, a total of 14 subpopulations were annotated using CD45 (PTPRC) (Suppl. Fig. S1E) and immune cell subsets 0, 1, 3, 6, 7, 10 and 13 were evaluated.

**Figure 1:**
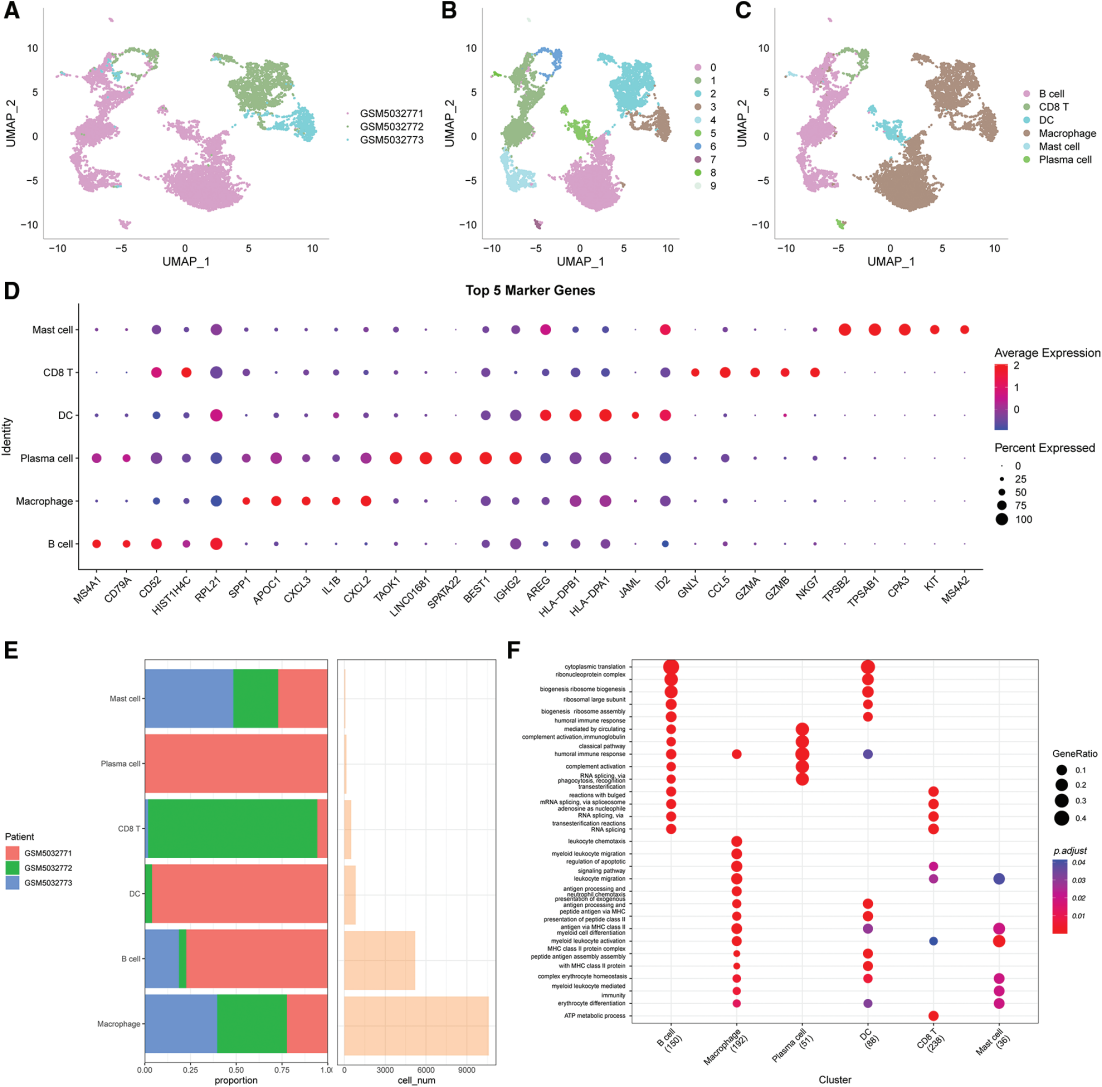
Clustering and dimensionality reduction analysis of single-cell data and enrichment analysis of annotated subgroups. (A) UMAP plot of the distribution of the 3 samples, (B) UMAP plot of the 10 immune cells subpopulations, (C) UMAP plot of the cell distribution after annotation, (D) dot plots showing the expression levels of the top 5 marker genes in the annotated subpopulations, (E) the proportion and number of cells of the annotated subpopulations in each sample, (F) results of GO-BP enrichment analysis of the annotated subpopulations.

The immune cells were extracted, re-Clustered, then further evaluated to obtain 10 distinct subpopulations. UMAP dimensionality reduction analysis was conducted for 9884 cells and the data were annotated by classical marker. Subpopulation 6 for CD8 T cells (expressing CD3D and GZMA). Subpopulations 1, 4 and 9 comprised B cells (expressing MS4A1, CD19 and CD79); subpopulation 7 comprised plasma cells (expressing CD79A and JSRP1); subpopulation 8 consisted of mast cells expressing (TPSAB1 and CPA3); subpopulation 0, 2 and 3 consisted of macrophages (expressing CD163, CD68 and CD14); subpopulation 6 consisted of DC (expressing CD1C and CD1E) (Suppl. Fig. S2). A UMAP plot showing the distribution of the three samples is presented in Fig. 1A. A UMAP plot of the different subpopulations after Clustering is shown in Fig. 1B. A UMAP plot of the distribution of cells after annotation is presented in Fig. 1C.

The first five marker genes that significantly contributed to expression in each subgroup were selected (Fig. 1D). The findings for the marker genes are presented in Table scRNA_marker_gene.txt.

The proportions of each sample for these 6 subpopulations were determined (Fig. 1E) and BP enrichment analyses were performed using the “ClusterProfiler” package (Fig. 1F).

### Immunologic dysregulation in single-cell TME

**Figure 2:**
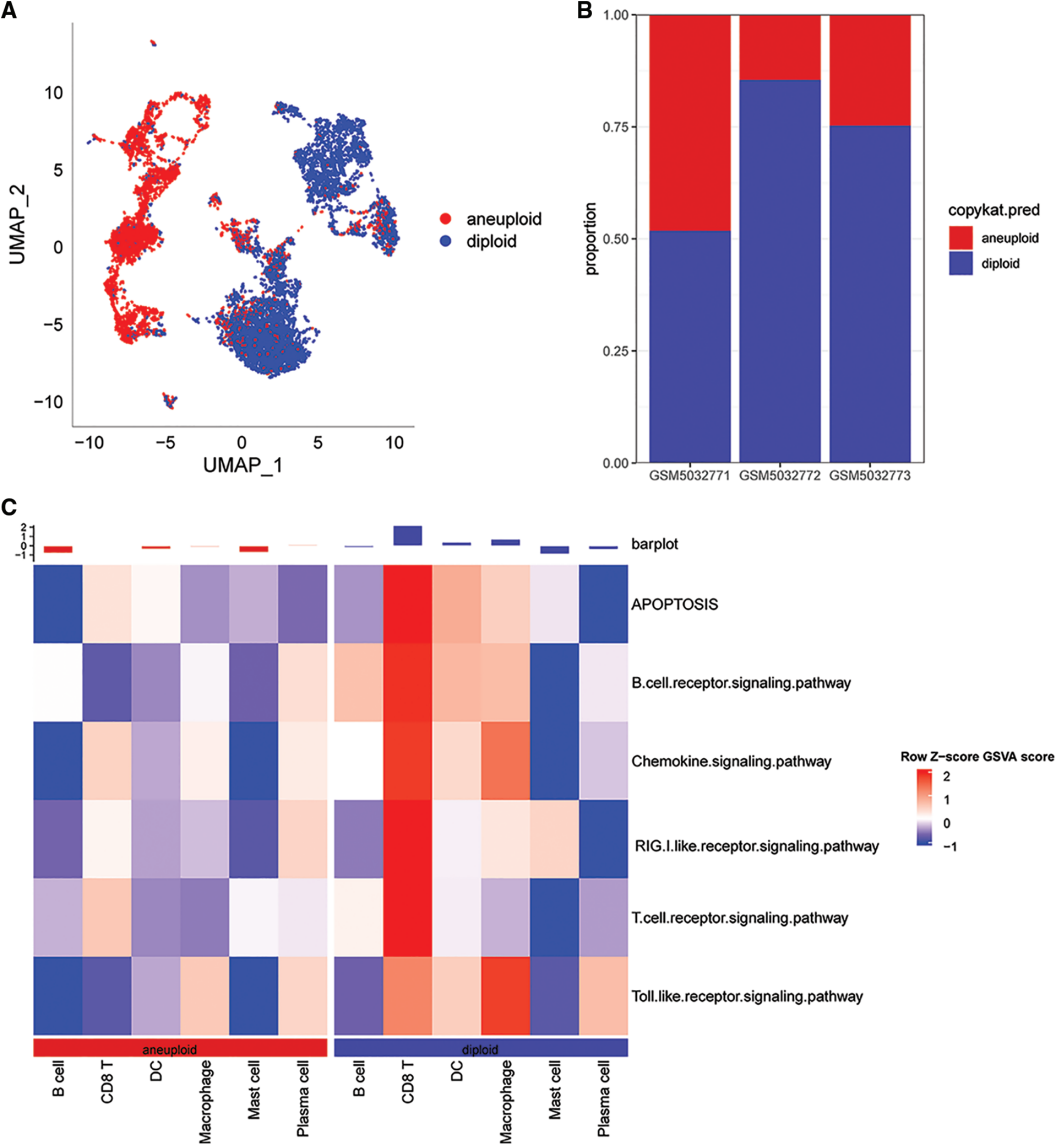
Distribution of malignant and non-malignant cells in single cell data and pathway scores associated with cGAS.STING. (A) UMAP plot using the copykat package for predicting the distribution of malignant and non-malignant cells; (B) proportion of malignant and non-malignant cells per sample; (C) cGAS.STING-related pathway scores correlated with malignant.

In this study, 6,812 cancer cells and 10,536 normal cells were identified. A UMAP diagram was generated using the copykat package to distinguish between normal and tumor cells (Fig. 2A). The proportion of non-malignant cells (diploid) and malignant cells (aneuploid) in each sample was evaluated (Fig. 2B).

The results showed that the correlation scores for cGAS.STING were lower in in CD8T cells for the malignant cells compared with the non-malignant cells (Fig. 2C).

### Key genes were identified through bulk RNA-seq

**Figure 3:**
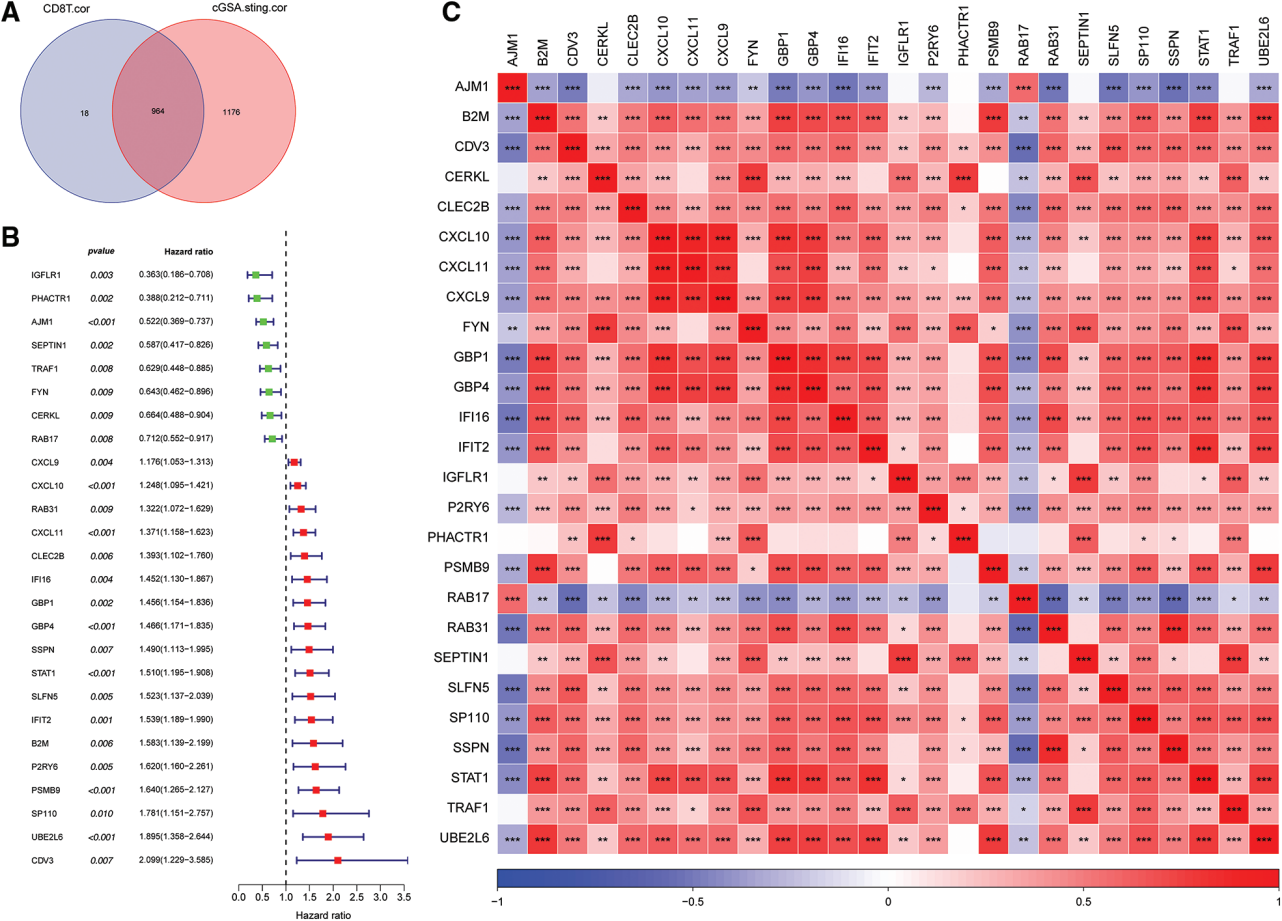
Screening for cGAS.STING-related genes and gene correlation analysis. (A) Venn plot of cGAS.STING-related genes and CD8 T cell-related genes; (B) forest plot of single factor cox analysis of prognostic-related genes; (C) heat map of prognostic-related gene correlation analysis.

A total of 964 key genes associated with cGAS.STING and CD8 T cells were obtained from the previous analysis (Fig. 3A).

Univariate Cox analysis of the 964 genes revealed that 26 genes were significantly correlated with survival of prostate cancer patients (Fig. 3B). Pearson correlation analysis of the 26 prognosis-related genes indicated that they were significantly correlated with each other (Fig. 3C).

### Identification of molecular subtypes based on CD8 T cells and cGAS.SING-related genes

**Figure 4:**
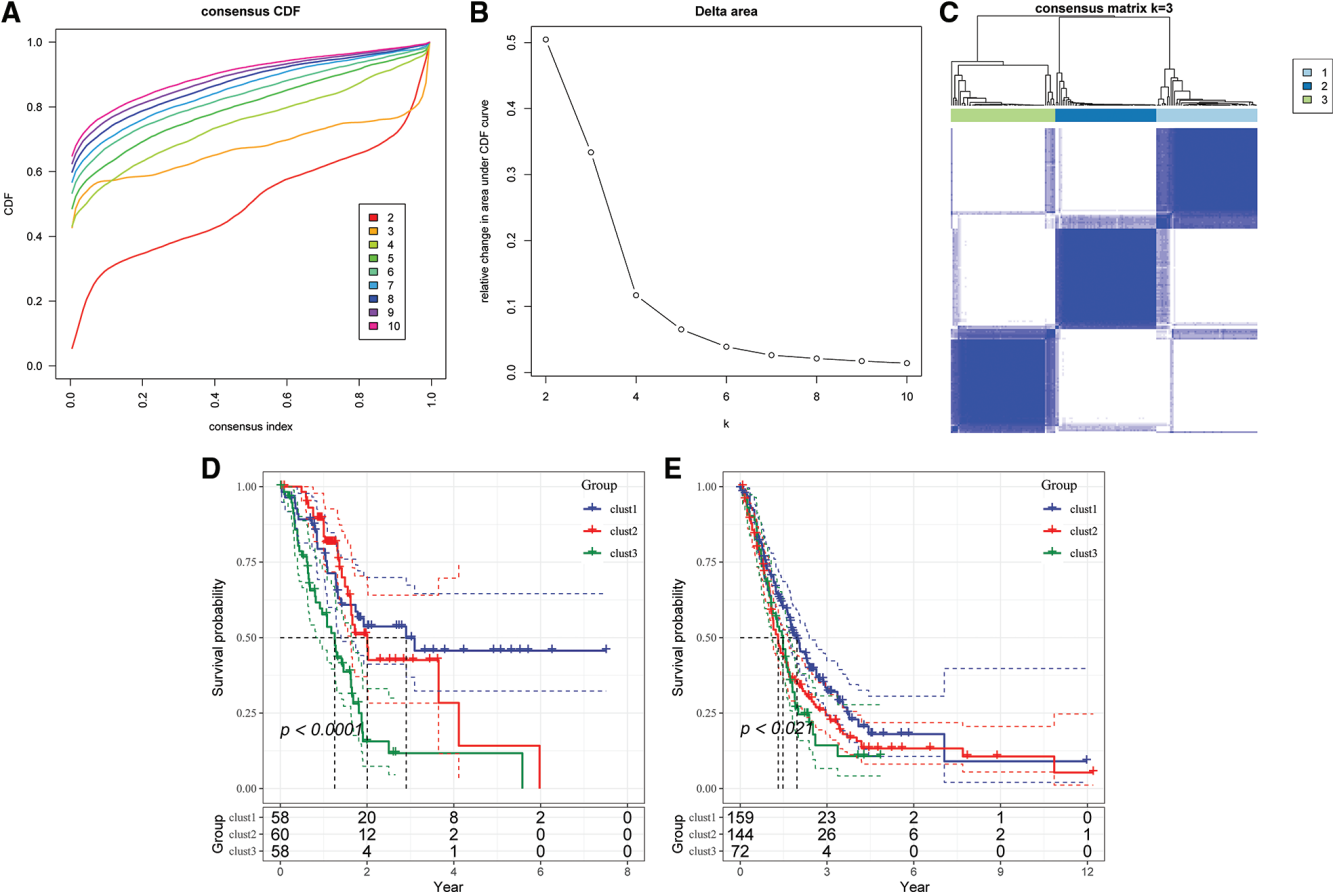
Construction of molecular subtypes. (A) TCGA cohort sample CDF curve; (B) TCGA cohort sample CDF Delta area curve, Delta area curve of consensus Clustering, indicating the relative change in area under the cumulative distribution function (CDF) curve for each category number k compared with k – 1. The horizontal axis represents the category number k and the vertical axis represents the relative change in area under CDF curve; (C) heat map of sample Clustering when consensus k = 3; (D) KM curve of the relationship between the three subtypes of TCGA; (E) prognostic KM curves of the three subtypes in the combined GEO cohort.

The optimum number of Clusters was determined based on the cumulative distribution function (CDF). The CDF delta area curve showed that the Clustering results were more stable when the Cluster size was chosen as 3 (Figs. 4A and 4B), so k = 3 was used to obtain the three molecular subtypes (Fig. 4C). Further analysis of the prognostic features for the three molecular subtypes indicated distinct prognostic differences among the subtypes (Fig. 4D). Clust3 had the worst prognosis, followed by Clust2, whereas Clust1 had the best prognosis. Table tcga.subtype.txt shows the data for the TCGA dataset subtypes. Fig. 4E shows the prognostic relational KM curves of the three subtypes in the combined GEO cohort.

PCA was conducted after renormalization of data to view the distribution of GEO data before and after the batch was removed (Suppl. Fig. S3). The results showed that the data sets of each sample after the batch removal were Clustered together, indicating that there was no batch effect in the merged data.

### Comparison of clinical phenotypes of the molecular subtypes

**Figure 5:**
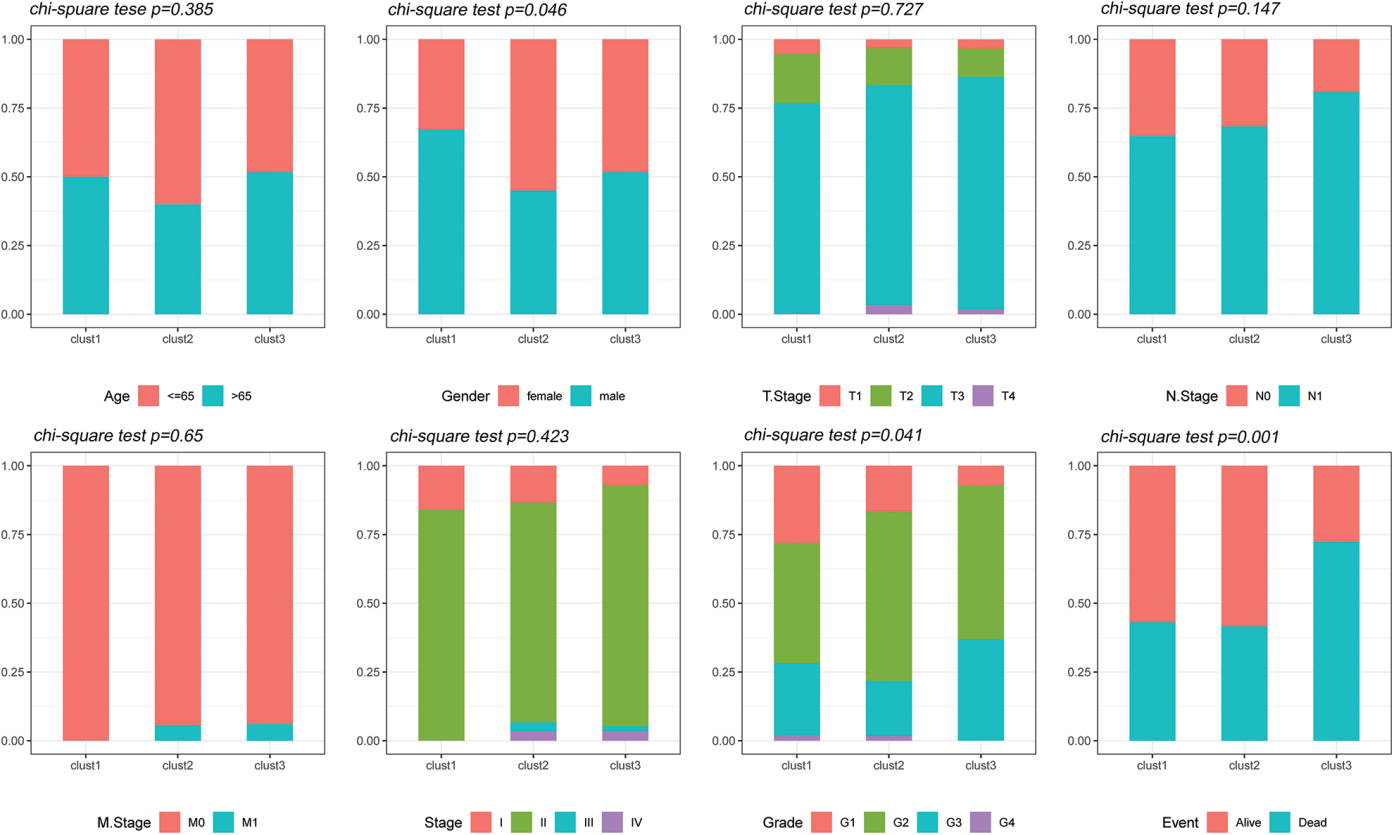
Clinical features of molecular subtypes. Comparison of the distribution of the clinical characteristics of the three molecular subtypes in the TCGA dataset.

The distribution of the various clinical features in the three molecular subtypes from the TCGA dataset was compared to determine whether the clinical characteristics differed among the different subtypes (chi-square test). The results revealed significant differences in gender, tumor grade and survival status between the three subtypes in the TCGA dataset (Fig. 5).

### Mutational characteristics of molecular subtypes

**Figure 6:**
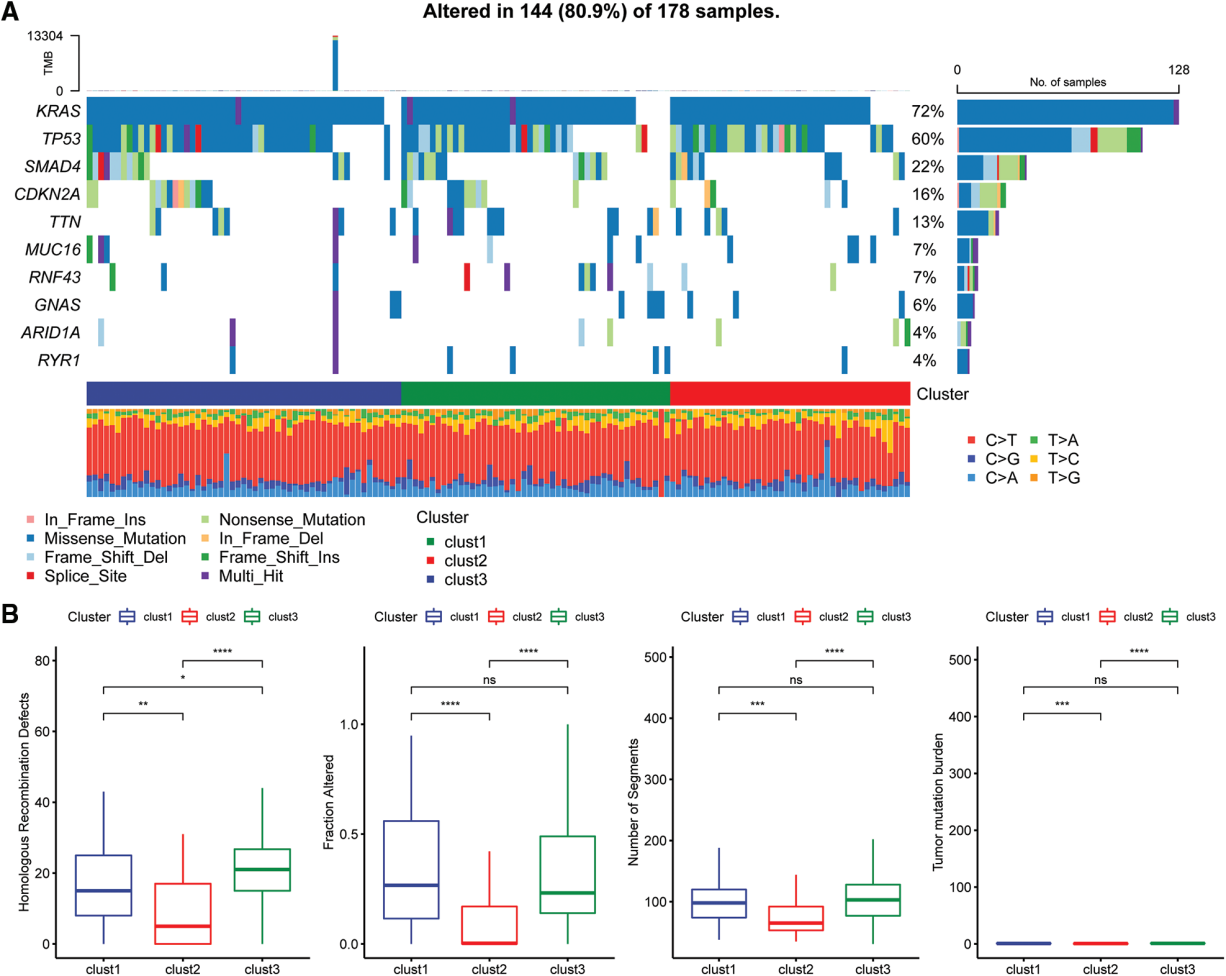
Genomic alterations in molecular subtypes of the TCGA cohort. (A) Somatic mutation analysis of different molecular subtypes in the TCGA cohort; (B) comparison of homologous recombination defects, fraction altered, number of segments, and tumor mutation burden among the different molecular subtypes of the TCGA cohort.

The “SNV” mutation data were obtained from TCGA database using “Mutect2”. The top 10 genes with the most significant mutations in each subtype are presented in Fig. 6A. The results indicated that Fraction Altered, homologous recombination defects, tumor mutation burden and number of segments were significantly different between the various subtypes (Fig. 6B).

### Immunologic characteristics of the molecular subtypes and differences between chemotherapy and immunotherapy

**Figure 7:**
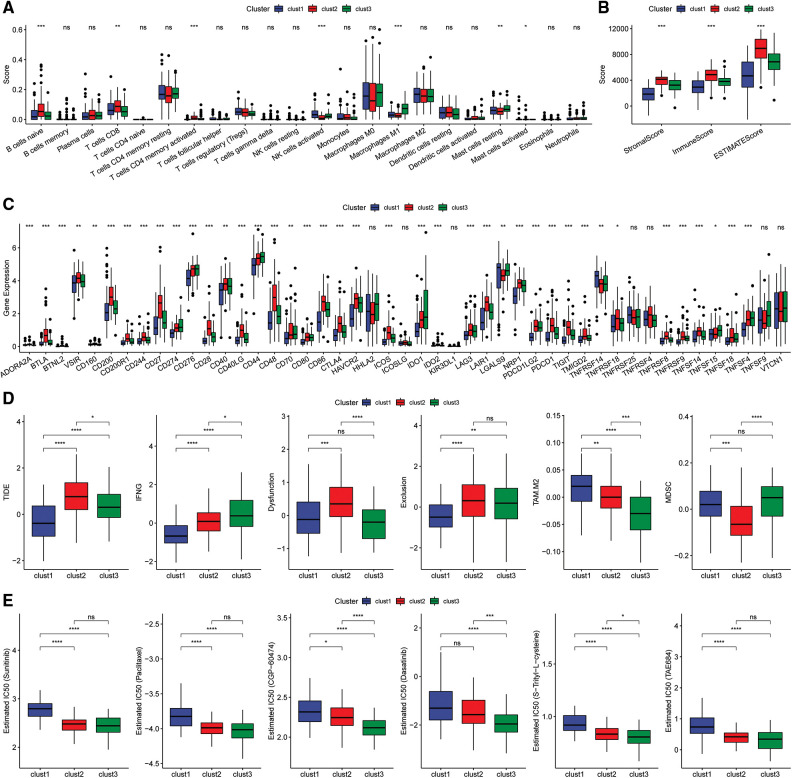
Immunoassay of molecular subtypes. (A) Differences in scores of the 22 immune cells among the different molecular subtypes in the TCGA cohort; (B) differences in ESTIMATE immune infiltration between the different molecular subtypes in the TCGA cohort; (C) differential expression of the immune checkpoint genes among the different molecular subtypes in the TCGA cohort; (D) differences in TIDE scores among the dif ferent molecular subtypes in the TCGA cohort; (E) box plots showing the estimated IC50 for drugs in TCGA-PAAD.

The scores of 22 immune cells were determined with the “CIBERSORT” algorithm and a “Kruskal.test” was conducted to evaluate the differences in scores among the three subtypes (Fig. 7A). The immune cell infiltration levels were assessed using the ESTIMATE tool (Fig. 7B). The findings indicated that the “ImmuneScore” was highest for the Clust2 subtype, whereas that of Clust1 was lowest. The expression levels of immune checkpoint genes were evaluated in three subtypes and significant differences in expression levels were observed between the three subtypes (Fig. 7C).

Analysis of the differences in response to immunotherapy of the various subtypes revealed that the TIDE score for the Clust1 subtype was lower than in Clust2 and Clust3 in the TCGA cohort (Fig. 7D). In addition, the response level of various molecular subtypes to conventional chemotherapeutic agents in TCGA cohort was evaluated. The results indicated that Clust3 was more sensitive to the chemotherapy agents compared with Clust1 and Clust2 (Fig. 7E, Wilcoxon test).

### Pathway analysis of molecular subtypes

**Figure 8:**
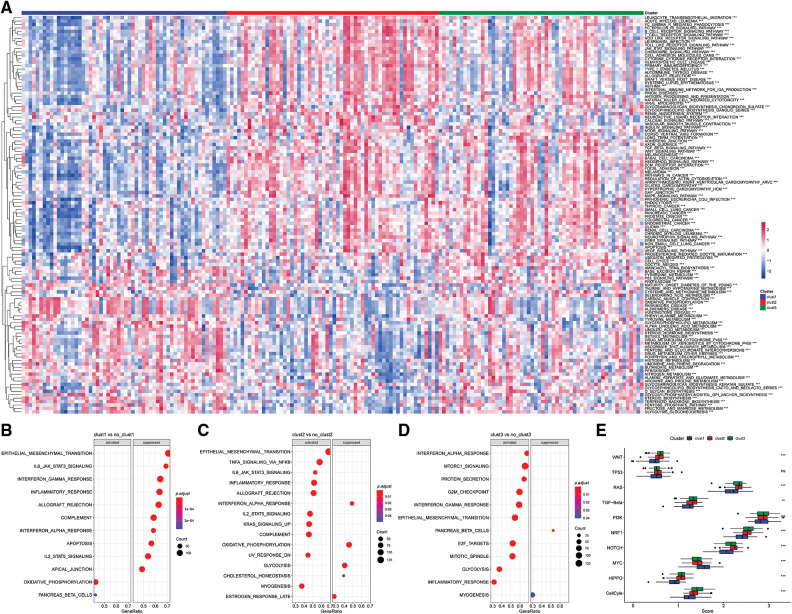
Pathway analysis of molecular subtypes. (A) Heat map of enrichment scores of kegg-related pathways in the three subtypes; (B) bubble plot of Clust1 activated/inhibited related pathways in the comparison of Clust1 *vs*. no_Clust1 subtypes; (C) comparison of Clust2 *vs*. no_Clust2 subtypes middle, the bubble diagram of the pathways related to the activation/inhibition of Clust2; (D) the bubble diagram of the pathways related to the activation/inhibition of Clust3 in the comparison of Clust3 *vs*. no_Clust3 subtypes; (E) different molecular subtypes in the 10 pathways related to tumor abnormalities scoring difference.

The “kruskal.test” function was used to determine the significance of the pathway score among for the three subtypes, and key pathways were determined using *p* < 0.001 (Fig. 8A).

The results showed significant enrichment of the OXIDATIVE_PHOSPHORYLATION and PANCREAS_BETA_CELLS pathway in Clust1 subtype, whereas IL6/JAK/STAT3 signaling pathway was significantly downregulated compared with the other Clusters. The IL6/JAK/STAT3 signaling pathway was significantly enriched in Clust2 subtype compared with the other Clusters, whereas the INTERFERON_ALPHA_RESPONSE pathway was downregulated. The INTERFERON_GAMMA_RESPONSE pathway was significantly upregulated in Clust3 subtype, whereas PANCREAS_BETA_CELLS pathway was downregulated compared with the other subtypes (Figs. 8B–8D).

Further, of the differences in 10 previously reported oncogenic pathways were evaluated among the three molecular subtypes. The findings showed significant differences in the evaluated pathways except he TP53 and PI3K pathways (Fig. 8E, Kruskal test).

### Identification of key genes and construction of risk models for CD8 T cells and cGAS.STING phenotype

In this study, 15 up-regulated genes and 225 down-regulated genes were identified by comparison of Clust1 with the other Clusters. Further, 175 up-regulated genes, 11 down-regulated genes were identified after comparison of Clust2 with the other two Clusters. A total of 21 up-regulated genes and 9 down-regulated genes were identified by the comparison between Clust3 and the other Clusters. The details differential expression of genes are shown in Table tcga.subtype.c1vsno_c1.txt, tcga.subtype.c2vsno_c2.txt and tcga.subtype.c3vsno_c3.txt. In this study, 348 differentially expressed genes were identified as shown in Table com_gene.txt.

Univariate cox analysis was conducted for the 348 differentially expressed genes using the “survival” package and 60 prognosis-related genes were selected with *p* < 0.01 as the cut-off.

**Figure 9:**
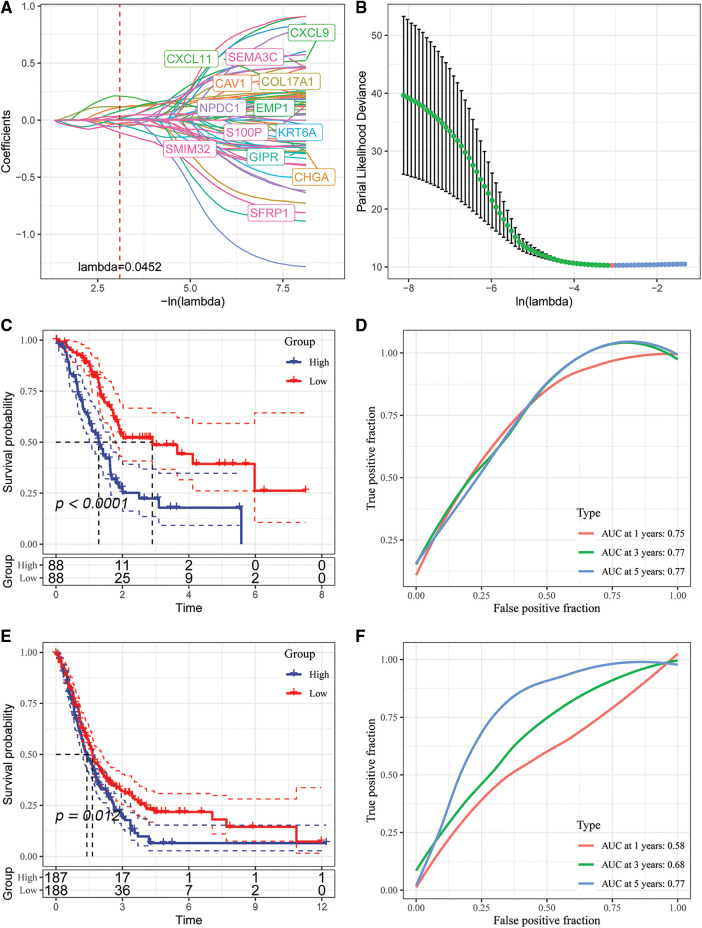
Identification of key genes and construction of risk models. (A) Individual independent variable trajectories with Lambda; (B) intervals of confidence under Lambda; (C) KM curves of risk models constructed from the TCGA data set of 5 genes, high or low risk; (D) ROC curve for the risk model developed using the five genes from the TCGA dataset.; (E) the five genes in the GSE dataset were used to build a risk model, and its KM curve is shown.; (F) ROC curve of the risk model developed using 5 genes from the GSE dataset.

Lasso regression was conducted to determine the independent genes associated with the prognosis of patients from the 60 prognosis-related genes in TCGA dataset to construct a risk model. The trajectory of each independent variable was evaluated as shown in Fig. 9A. The results showed that the number of independent variable coefficients decreased with increase in lambda variable. A 10-fold cross validation was used for model validation and the confidence interval under each variable was determined, confidence intervals are shown in Fig. 9B. In this study, 13 genes were selected as the next target genes when lambda was 0.0452. Stepwise multivariate regression analysis was conducted based on the 13 genes obtained from the lasso analysis. EMP1, GIPR, SFRP1, CXCL11 and COL17A1 were identified as the prognosis-related genes for construction of a prognostic signature. The final five-gene signature formula was as follows: Riskscore = 0.355 * EMP1-0.267 * GIPR-0.242 * SFRP1 + 0.28 * CXCL11 + 0.184 * COL17A1.

We analyzed the classification efficiency of prognostic prediction at 1, 3, 5 years, respectively, where the AUC reached 0.7 at 1, 3, 5 years, and also zscore the Riskscore, classify the samples with Riskscore greater than zero as high risk group and those with less than zero as low risk group after zscoreization, and plot KM curves, and found that they had highly significant differences *p* < 0.0001 (Figs. 9C and 9D).

To better validate the robustness of the model, we used the merged GSE dataset to validate using the same method, and similar results were obtained (Figs. 9E and 9F).

### Comparison of Riskscore with different clinicopathologic features

**Figure 10:**
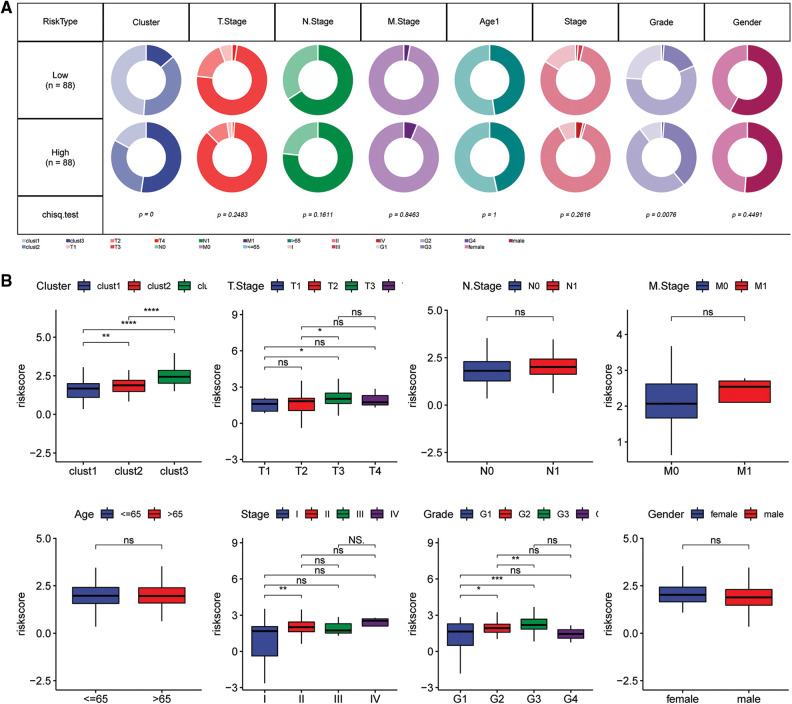
Clinical characteristics of the Riskscore score in the TCGA cohort. (A) Clinical phenotypes of the Riskscore subgroups in the TCGA cohort. (B) The difference in the Riskscore of various phenotypes in the TCGA cohort (Wilcox test, *p < 0.05; **p < 0.01; ***p < 0.001; and ****p < 0.0001).

Comparisons of differences in the clinicopathological characteristics between the Riskscore subgroups in the TCGA cohort showed similar results (Fig. 10A). The findings indicated that the risk score increase with increase in clinical grade (Fig. 10B).

### Combination of Riskscore with clinicopathological characteristics further improved the survival prediction of the constructed model

**Figure 11:**
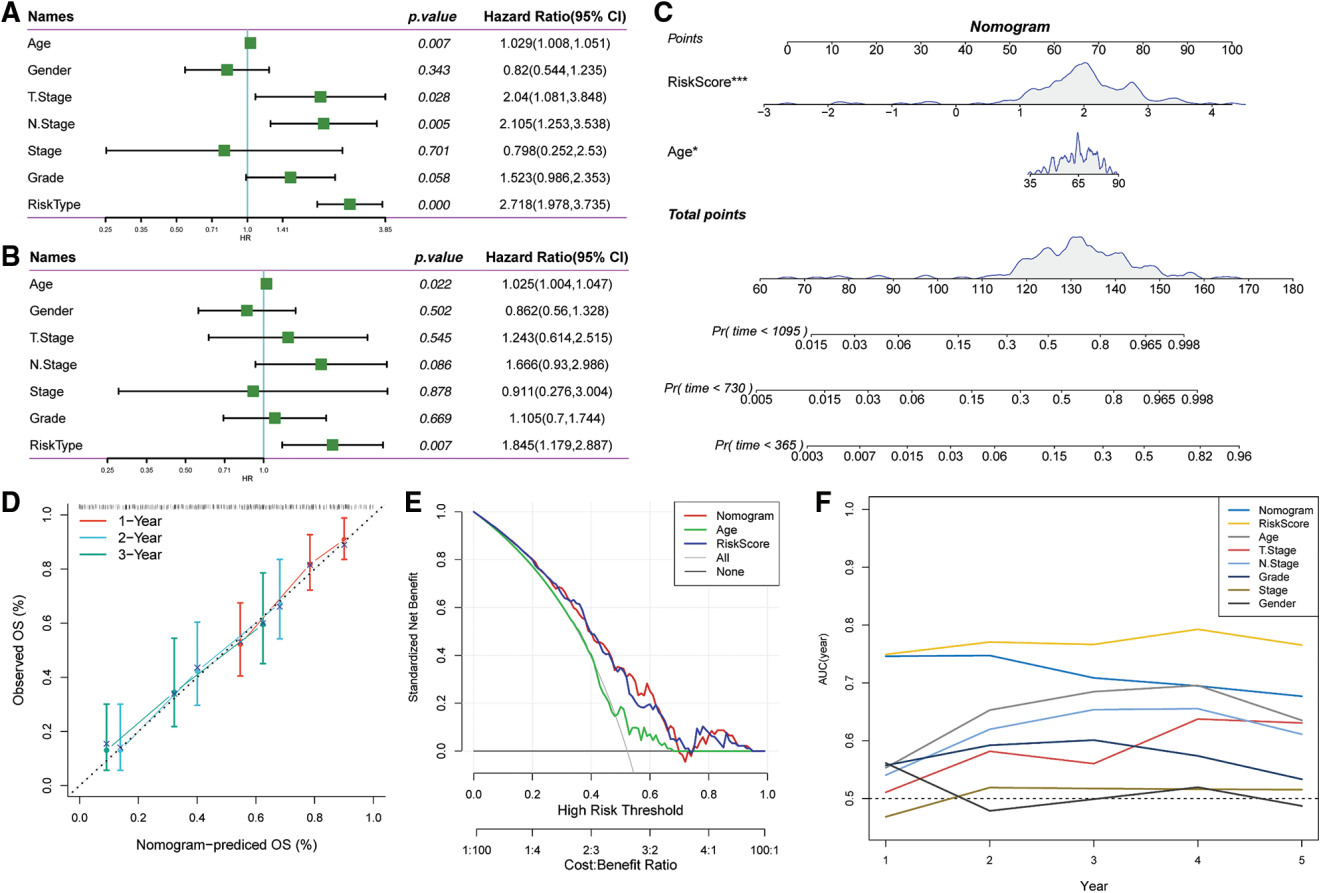
Prognostic modeling and survival prediction by Riskscore score and Nomogram. (A) Riskscore and clinical features obtained through single-variable Cox analysis; (B) cox analysis with several variables for clinical features and Riskscore; (C) nomogram model; (D) survival curve calibration for 1, 2, and 3 years; (E) The nomogram’s decision curve; (F) the nomogram had the highest ability to predict patient survival compared with other clinicopathological characteristics.

Univariate and multivariate Cox regression analyses of the clinical characteristics and Riskscore demonstrated that Riskscore was the most significant prognostic factor (Figs. 11A and 11B). We combined the Riskscore with other clinicopathologic features and established a nomogram to quantify risk evaluation and probability of survival of patients (Fig. 11C). Riskscore had the greatest impact on survival prediction. Calibration curves were generated to assess the model's prediction accuracy (Fig. 11D). The findings revealed that the calibration and prediction curves for three calibration points at 1, 2 and 3 years almost overlapped with the standard curve, indicating that the nomogram had good predictive performance. Evaluation of the reliability of model with a decision curve analysis (DCA) revealed that the survival prediction accuracy of the nomogram and Riskscore was significantly higher compared with the clinicopathological characteristics (Figs. 11E and 11F).

### qRT-PCR

**Figure 12:**
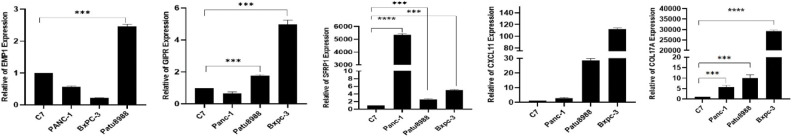
Expression levels of EMP1, GIPR, SFRP1, CXCL11and COL17A in normal and pancreatic cancer cells.

We performed qRT-PCR to verify the bioinformatics results. The experimental results showed that the mRNA expression levels of EMP1, GIPR, SFRP1, CXCL11 and COL17A were significantly high in the pancreatic cancer cell line compared with the controls. In this study, COL17A demonstrated the most significant expression difference between the samples. These findings indicate that the results obtained through bioinformatics analysis were reliable and have potential research value (Fig. 12).

## Discussion

The treatment of pancreatic cancer is mainly based on the histopathologic difference between its subtypes, but the results have been unsatisfactory. Significant progress has been achieved in the molecular typing of cancer in recent years owing to the rapid advances in high-throughput sequencing and single-cell sequencing. Molecular typing is effective in elucidating the occurrence and progression of tumors and development of individualized treatment. In this study, we found that the correlation score of cGAS.STING was lower in malignant cells than in non-malignant cells in CD8T cells. As a result, 26 key genes were identified and pancreatic cancer was grouped into three molecular subtypes. KM survival curves of the three subtypes indicated that Clust1 subtype had the highest prognosis compared with the other subtypes.

The conventional treatment approaches mainly used for pancreatic cancer include surgery, chemotherapy and neoadjuvant chemotherapy. Most pancreatic cancer patients are not eligible for curative surgical treatment during diagnosis because most cases are diagnosed at advanced stages. Immunotherapy is effective in treating pancreatic cancer. Therefore, we analyzed the immune characteristics of the three molecular subtypes, and the results showed that Clust1 had the lowest ImmuneScore. TIDE scores were lower for the Clust1 subtype compared with lust2 and Clust3, indicating that the Clust1 subtype had a higher risk of immune escape and had higher potential response to immunotherapy. These findings provide potential avenues for the treatment of pancreatic cancer. Notably, Cluster3(C3) was more sensitive to conventional chemotherapeutic drugs, indicating that different therapeutic regimens can be used for the different molecular types to improve treatment outcomes.

Differentially expressed genes between the three subtypes were evaluated and EMP1, GIPR, SFRP1, CXCL11 and COL17A1 were used to establish risk prediction and prognostic models. Analysis of the TCGA database showed that these five genes were significantly associated with the survival of patients. qRT-PCR showed a high expression levels of these five genes in pancreatic carcinoma.

EMP1 is a member of peripheral myelin protein 22-kDa (PMP22) gene family. Dysregulation of EMP1 expression is linked to epithelial diseases, particularly human cancers. EMP1 is mainly expressed in the early and immature neurons [27] and glioma [28], and in sclerotic gastric carcinoma cells [29]. EMP1 expression level can be used to to differentiate between invasive ductal and lobular breast carcinoma. This gene is associated with the ability of breast cancer to metastasize *in vitro* [30,31]. Microarray analysis showed that EMP1 was up-regulated by Her-2 in cDNA [32]. EMP1 has also is a bio-marker for HER-2 activation in breast cancer and is associated with lymph node metastasis in squamous cell carcinoma of the oral cavity [33]. EMP1 is correlated with clinical resistance to gefitinib in lung cancer [34]. Further, it is associated with *in vitro* resistance to prednisolone in leukemia cells [35]. High EMP1 expression level is associated with a low five-year event-free survival rate in precursor-B ALL patients [36].

Glucose-dependent insulin-stimulating peptide (GIP) is an insulin-stimulating hormone produced in the digestive tract and its release is induced by food intake [37]. It is a 42-amino acid peptide hormone previously associated with the suppression of gastric acid secretion [38]. It is mainly released in K cells of proximal small intestine (jejunum and duodenum) [39]. The main metabolic function of GIP is stimulating glucose-dependent pancreatic β cells to release insulin [40]. GIPR expression is dysregulated in several transformed endocrine tissues and may play a role in the etiopathogenesis of hormone secretion disorders [41].

SFRP1 is a member of the cysteine-rich structural domain of the SFRP family proteins. The cysteine-rich structural domain mimics the Wnt binding site of the coiled-coil receptor [42]. Proteins of the SFRP family modulate the Wnt signaling. SFRP1 inhibits WNT-dependent transcription and reduces intracellular β-catenin levels [43]. Several studies report that low expression level of SFRP1 is correlated with a poor prognosis in cholangiocarcinoma, lung, breast as well as hepatocellular carcinoma subjects [43–46].

Chemokine (C-X-C motif) ligand 11 (CXCL11), also known as interferon-inducible protein 9 (IP-9) or interferon-induced T-cell alpha chemokine (I-TAC), is a complex cytokine with many functions in different tumors. It is mainly expressed in the thymus, pancreas, lung, liver, spleen and peripheral leukocytes, but low expression levels are observed in the intestine, placenta and prostate [47]. CXCL11 promotes recruitment of activated NK, CTL and Th1 cells in the tumor tissue *in vivo* [48,49]. CXCL11 increases the frequency of tumor-infiltrating lymphocytes and inhibits tumor growth in both breast cancer and T-cell lymphoma [49–51]. CXCL11 exhibits potent anti-tumor activity *in vivo* by promoting CD8+ T lymphocyte infiltration. CXCL11 is positively correlated with tumor-infiltrating CD8+ T cells in mice subjected to a transgenic administered with CXCL11-EL4 t-cell lymphoma cells. CD8+T cell downregulation is associated with loss of CXCL11 antitumor effect *in vivo*. Upregulation of CXCL11 expression in EL4 cells transduced with CXCL11 promotes infiltration of total CD8+ and CD8+ CXCR3+ T lymphocyte and macrophages, with no impact on angiogenesis in EL4-CXCL11 tumors. This study demonstrated that local release of CXCL11 induces systemic tumor immunity [50,51].

Collagen XVII is an adhesive protein present in the basal epithelium. This protein is encoded by the COL17A1 gene and it modulates the growth and migration of cells [52]. COL17A1 is associated with a poor prognosis in some tumors [53,54]. Thangavelu et al. reported that hypomethylation and high expression of COL17A1 are correlated with poor prognosis in epithelial carcinoma [55]. Yan et al. observed that XVII collagen promoted aggressiveness and recurrence of gliomas [56]. The present findings showed a negative association between mRNA expression and DNA methylation of COL17A1 and a significant increase in CNV of COL17A1. Furthermore, COL17A1 expression is modulated by CNV and DNA methylation. GSEA was conducted to explore the mechanism of action of COL17A1 in PC. The findings revealed that COL17A1 was correlated with the development of epithelial cells and the cell cycle. The transformation from monolayer to multilayer epithelial structures is a key marker of carcinogenesis [57], indicating that COL17A1 is an essential factor in the development and maintenance of multilayered epithelial structures [57]. These findings indicate that COL17A1 is implicated in the progression of PC by modulating the proliferation and differentiation of epithelial cells.

The KM survival curve showed significant differences between the molecular subtypes. The model’s accuracy in clinical prognosis was evaluated by merging the base and generating KM survival curves. The prognostic model was combined with various clinicopathological characteristics to enhance its clinical significance. The findings revealed that that the constructed nomogram and Riskscore had the highest survival prediction ability.

The present study was based on the retrieval and reanalysis of samples from public databases, thus the findings are dependent on the quality, accuracy and completeness of the obtained samples. Furthermore, some genes were not available in the meta-cohort because analysis required comparison of different techniques for gene expressions quantification. However, the effect of deletion on subtype prediction was negligible as shown by the good molecular characteristics of the pancreatic cancer subtypes consistent with other previously reported subtypes. The clinical relevance of the three molecular subtypes is based on analysis of survival data. The treatment effect of these subtypes was not evaluated due to lack of treatment-related data.

## Conclusion

In this study, we applied a bioinformatics approach based on CGAS.STING to classify pancreatic cancer into three molecular subtypes. We evaluated the differences in immunological characteristics, immunotherapy, chemotherapy and molecular pathways of the three molecular subtypes. Five genes were selected and used to build a prognostic risk model, which showed high clinical prognostic performance.

## Supplementary Materials

Table of genes differing between subtypes tcga.subtype.c1vsno_c1.txt
tcga.subtype.c2vsno_c2.txt
tcga.subtype.c3vsno_c3.txt

Data for TCGA dataset subtypes tcga.subtype.txt

Table of differential gene com_gene.txt

Table of results for marker genes scRNA_marker_gene.txt

FIGURE S1. Single cell clustering dimensionality reduction analysis.

FIGURE S2. UMAP plots of marker gene expression.

FIGURE S3. PCA analysis of each data set before and after exclusion of batches.

## Data Availability

Data reported in this paper are available from the corresponding author upon reasonable request.

## References

[1] Siegel, R. L., Miller, K. D., Fuchs, H. E., Jemal, A. (2021). Cancer statistics, 2021. CA: A Cancer Journal for Clinicians*,* 71 *(*1*),* 7–33. 10.3322/caac.21654; 33433946

[2] Mulkeen, A. L., Yoo, P. S., Cha, C. (2006). Less common neoplasms of the pancreas. World Journal of Gastroenterology*,* 12 *(*20*),* 3180–3185. 10.3748/wjg.v12.i20.3180; 16718837 PMC4087960

[3] Hackert, T., Werner, J., Weitz, J., Schmidt, J., Buchler, M. W. (2010). Uncinate process first–A novel approach for pancreatic head resection. Langenbeck’s Archives of Surgery*,* 395 *(*8*),* 1161–1164. 10.1007/s00423-010-0663-9; 20582600

[4] Sinha, V., Shinde, S., Saxena, S., Thakur, S., Walia, T. et al. (2020). A comprehensive review of diagnostic and therapeutic strategies for the management of pancreatic cancer. Critical Reviews in Oncogenesis*,* 25 *(*4*),* 381–404. 10.1615/CritRevOncog.2020035971; 33639064

[5] Heestand, G. M., Murphy, J. D., Lowy, A. M. (2015). Approach to patients with pancreatic cancer without detectable metastases. Journal of Clinical Oncology*,* 33 *(*16*),* 1770–1778. 10.1200/JCO.2014.59.7930; 25918279

[6] Strobel, O., Neoptolemos, J., Jager, D., Buchler, M. W. (2019). Optimizing the outcomes of pancreatic cancer surgery. Nature Reviews Clinical Oncology*,* 16 *(*1*),* 11–26. 10.1038/s41571-018-0112-1; 30341417

[7] Xu, J. W., Wang, L., Cheng, Y. G., Zhang, G. Y., Hu, S. Y. et al. (2018). Immunotherapy for pancreatic cancer: A long and hopeful journey. Cancer Letters*,* 425*,* 143–151. 10.1016/j.canlet.2018.03.040; 29605510

[8] Kim, J. M., Chen, D. S. (2016). Immune escape to PD-L1/PD-1 blockade: Seven steps to success (or failure). Annals of Oncology*,* 27 *(*8*),* 1492–1504. 10.1093/annonc/mdw217; 27207108

[9] Das, S., Berlin, J., Cardin, D. (2018). Harnessing the immune system in pancreatic cancer. Current Treatment Options in Oncology*,* 19 *(*10*),* 48. 10.1007/s11864-018-0566-5; 30128712 PMC6524529

[10] Collisson, E. A., Bailey, P., Chang, D. K., Biankin, A. V. (2019). Molecular subtypes of pancreatic cancer. Nature Reviews Gastroenterology & Hepatology*,* 16 *(*4*),* 207–220. 10.1038/s41575-019-0109-y; 30718832

[11] Barkley, D., Rao, A., Pour, M., Franca, G. S., Yanai, I. (2021). Cancer cell states and emergent properties of the dynamic tumor system. Genome Research*,* 31 *(*10*),* 1719–1727. 10.1101/gr.275308.121; 34599005 PMC8494223

[12] Connor, A. A., Denroche, R. E., Jang, G. H., Timms, L., Kalimuthu, S. N. et al. (2017). Association of distinct mutational signatures with correlates of increased immune activity in pancreatic ductal adenocarcinoma. JAMA Oncology*,* 3 *(*6*),* 774–783. 10.1001/jamaoncol.2016.3916; 27768182 PMC5824324

[13] Duell, E. J., Lucenteforte, E., Olson, S. H., Bracci, P. M., Li, D. et al. (2012). Pancreatitis and pancreatic cancer risk: A pooled analysis in the international pancreatic cancer case-control consortium (PanC4). Annals of Oncology*,* 23 *(*11*),* 2964–2970. 10.1093/annonc/mds140; 22767586 PMC3477881

[14] Lowenfels, A. B., Maisonneuve, P., Cavallini, G., Ammann, R. W., Lankisch, P. G. et al. (1993). Pancreatitis and the risk of pancreatic cancer. International pancreatitis study group. The New England Journal of Medicine*,* 328 *(*20*),* 1433–1437. 10.1056/NEJM199305203282001; 8479461

[15] Malka, D., Hammel, P., Maire, F., Rufat, P., Madeira, I. et al. (2002). Risk of pancreatic adenocarcinoma in chronic pancreatitis. Gut*,* 51 *(*6*),* 849–852. 10.1136/gut.51.6.849; 12427788 PMC1773474

[16] Munigala, S., Kanwal, F., Xian, H., Scherrer, J. F., Agarwal, B. (2014). Increased risk of pancreatic adenocarcinoma after acute pancreatitis. Clinical Gastroenterology and Hepatology*,* 12 *(*7*),* 1143–1150.e1141. 10.1016/j.cgh.2013.12.033; 24440214

[17] Bracci, P. M., Wang, F., Hassan, M. M., Gupta, S., Li, D. et al. (2009). Pancreatitis and pancreatic cancer in two large pooled case-control studies. Cancer Causes & Control*,* 20 *(*9*),* 1723–1731. 10.1007/s10552-009-9424-x; 19760029 PMC2767517

[18] Ahn, J., Xia, T., Konno, H., Konno, K., Ruiz, P. et al. (2014). Inflammation-driven carcinogenesis is mediated through STING. Nature Communications*,* 5 *(*1*),* 5166. 10.1038/ncomms6166; 25300616 PMC4998973

[19] Barbie, D. A., Tamayo, P., Boehm, J. S., Kim, S. Y., Moody, S. E. et al. (2009). Systematic RNA interference reveals that oncogenic KRAS-driven cancers require TBK1. Nature*,* 462 *(*7269*),* 108–112. 10.1038/nature08460; 19847166 PMC2783335

[20] Izar, B., Tirosh, I., Stover, E. H., Wakiro, I., Cuoco, M. S. et al. (2020). A single-cell landscape of high-grade serous ovarian cancer. Nature Medicine*,* 26 *(*8*),* 1271–1279. 10.1038/s41591-020-0926-0; 32572264 PMC7723336

[21] Fridman, W. H., Pages, F., Sautes-Fridman, C., Galon, J. (2012). The immune contexture in human tumours: Impact on clinical outcome. Nature Reviews Cancer*,* 12 *(*4*),* 298–306. 10.1038/nrc3245; 22419253

[22] Fridman, W. H., Miller, I., Sautes-Fridman, C., Byrne, A. T. (2020). Therapeutic targeting of the colorectal tumor stroma. Gastroenterology*,* 158 *(*2*),* 303–321. 10.1053/j.gastro.2019.09.045; 31622621

[23] Zhang, X., Shi, M., Chen, T., Zhang, B. (2020). Characterization of the immune cell infiltration landscape in head and neck squamous cell carcinoma to aid immunotherapy. Molecular Therapy Nucleic Acids*,* 22*,* 298–309. 10.1016/j.omtn.2020.08.030; 33230435 PMC7522342

[24] Arneth, B. (2019). Tumor microenvironment. Medicina*,* 56 *(*1*),* 15. 10.3390/medicina56010015; 31906017 PMC7023392

[25] Yang, K. S., Xu, C. Q., Lv, J. (2021). Identification and validation of the prognostic value of cyclic GMP-AMP synthase-stimulator of interferon (cGAS-STING) related genes in gastric cancer. Bioengineered*,* 12 *(*1*),* 1238–1250. 10.1080/21655979.2021.1911557; 33843442 PMC8291813

[26] Sanchez-Vega, F., Mina, M., Armenia, J., Chatila, W. K., Luna, A. et al. (2018). Oncogenic signaling pathways in the cancer genome atlas. Cell*,* 173 *(*2*),* 321–337.e310. 10.1016/j.cell.2018.03.035; 29625050 PMC6070353

[27] Wulf, P., Suter, U. (1999). Embryonic expression of epithelial membrane protein 1 in early neurons. Brain Research Developmental Brain Research*,* 116 *(*2*),* 169–180. 10.1016/S0165-3806(99)00092-9; 10521561

[28] Bredel, M., Bredel, C., Juric, D., Harsh, G. R., Vogel, H. et al. (2005). Functional network analysis reveals extended gliomagenesis pathway maps and three novel MYC-interacting genes in human gliomas. Cancer Research*,* 65 *(*19*),* 8679–8689. 10.1158/0008-5472.CAN-05-1204; 16204036

[29] Hippo, Y., Yashiro, M., Ishii, M., Taniguchi, H., Tsutsumi, S. et al. (2001). Differential gene expression profiles of scirrhous gastric cancer cells with high metastatic potential to peritoneum or lymph nodes. Cancer Research*,* 61 *(*3*),* 889–895; 11221876

[30] Gnirke, A. U., Weidle, U. H. (1998). Investigation of prevalence and regulation of expression of progression associated protein (PAP). Anticancer Research*,* 18 *(*6A*),* 4363–4369; 9891493

[31] Turashvili, G., Bouchal, J., Ehrmann, J., Fridman, E., Skarda, J. et al. (2007). Novel immunohistochemical markers for the differentiation of lobular and ductal invasive breast carcinomas. Biomedical Papers of the Medical Faculty of the University Palacky, Olomouc, Czechoslovakia*,* 151 *(*1*),* 59–64. 10.5507/bp.2007.010; 17690741

[32] Mackay, A., Jones, C., Dexter, T., Silva, R. L., Bulmer, K. et al. (2003). cDNA microarray analysis of genes associated with ERBB2 (HER2/neu) overexpression in human mammary luminal epithelial cells. Oncogene*,* 22 *(*17*),* 2680–2688. 10.1038/sj.onc.1206349; 12730682

[33] Zhang, J., Cao, W., Xu, Q., Chen, W. T. (2011). The expression of EMP1 is downregulated in oral squamous cell carcinoma and possibly associated with tumour metastasis. Journal of Clinical Pathology*,* 64 *(*1*),* 25–29. 10.1136/jcp.2010.082404; 20980531

[34] Jain, A., Tindell, C. A., Laux, I., Hunter, J. B., Curran, J. et al. (2005). Epithelial membrane protein-1 is a biomarker of gefitinib resistance. Proceedings of the National Academy of Sciences of the United States of America*,* 102 *(*33*),* 11858–11863. 10.1073/pnas.0502113102; 16087880 PMC1187965

[35] Yu, Y. H., Kuo, H. K., Chang, K. W. (2008). The evolving transcriptome of head and neck squamous cell carcinoma: A systematic review. PLoS One*,* 3 *(*9*),* e3215. 10.1371/journal.pone.0003215; 18791647 PMC2533097

[36] Aries, I. M., Jerchel, I. S., van den Dungen, R. E., van den Berk, L. C., Boer, J. M. et al. (2014). EMP1, a novel poor prognostic factor in pediatric leukemia regulates prednisolone resistance, cell proliferation, migration and adhesion. Leukemia*,* 28 *(*9*),* 1828–1837. 10.1038/leu.2014.80; 24625531

[37] Baggio, L. L., Drucker, D. J. (2007). Biology of incretins: GLP-1 and GIP. Gastroenterology*,* 132 *(*6*),* 2131–2157. 10.1053/j.gastro.2007.03.054; 17498508

[38] Shimosegawa, T., Toyota, T. (1999). Gastric inhibitory polypeptide (GIP). Nihon Rinsho Japanese Journal of Clinical Medicine*,* S57*,* 357–359. 10778138

[39] Mortensen, K., Christensen, L. L., Holst, J. J., Orskov, C. (2003). GLP-1 and GIP are colocalized in a subset of endocrine cells in the small intestine. Regulatory Peptides*,* 114 *(*2–3*),* 189–196. 10.1016/S0167-0115(03)00125-3; 12832109

[40] Dupre, J., Ross, S. A., Watson, D., Brown, J. C. (1973). Stimulation of insulin secretion by gastric inhibitory polypeptide in man. The Journal of Clinical Endocrinology and Metabolism*,* 37 *(*5*),* 826–828. 10.1210/jcem-37-5-826; 4749457

[41] Regazzo, D., Barbot, M., Scaroni, C., Albiger, N., Occhi, G. (2020). The pathogenic role of the GIP/GIPR axis in human endocrine tumors: Emerging clinical mechanisms beyond diabetes. Reviews in Endocrine & Metabolic Disorders*,* 21 *(*1*),* 165–183. 10.1007/s11154-019-09536-6; 31933128

[42] Pei, J., Grishin, N. V. (2012). Cysteine-rich domains related to frizzled receptors and hedgehog-interacting proteins. Protein Science*,* 21 *(*8*),* 1172–1184. 10.1002/pro.2105; 22693159 PMC3537238

[43] Esteve, P., Bovolenta, P. (2010). The advantages and disadvantages of sfrp1 and sfrp2 expression in pathological events. The Tohoku Journal of Experimental Medicine*,* 221 *(*1*),* 11–17. 10.1620/tjem.221.11; 20448436

[44] Ren, J., Wang, R., Huang, G., Song, H., Chen, Y. et al. (2013). sFRP1 inhibits epithelial-mesenchymal transition in A549 human lung adenocarcinoma cell line. Cancer Biotherapy & Radiopharmaceuticals*,* 28 *(*7*),* 565–571. 10.1089/cbr.2012.1453; 23802127 PMC3741431

[45] Sur, S., Pal, D., Mandal, S., Roy, A., Panda, C. K. (2016). Tea polyphenols epigallocatechin gallete and theaflavin restrict mouse liver carcinogenesis through modulation of self-renewal Wnt and hedgehog pathways. The Journal of Nutritional Biochemistry*,* 27 *(*19*),* 32–42. 10.1016/j.jnutbio.2015.08.016; 26386739

[46] Bernemann, C., Hulsewig, C., Ruckert, C., Schafer, S., Blumel, L. et al. (2014). Influence of secreted frizzled receptor protein 1 (SFRP1) on neoadjuvant chemotherapy in triple negative breast cancer does not rely on WNT signaling. Molecular Cancer*,* 13 *(*1*),* 174. 10.1186/1476-4598-13-174; 25033833 PMC4110378

[47] Cole, K. E., Strick, C. A., Paradis, T. J., Ogborne, K. T., Loetscher, M. et al. (1998). Interferon-inducible T cell alpha chemoattractant (I-TAC): A novel non-ELR CXC chemokine with potent activity on activated T cells through selective high affinity binding to CXCR3. The Journal of Experimental Medicine*,* 187 *(*12*),* 2009–2021. 10.1084/jem.187.12.2009; 9625760 PMC2212354

[48] Campanella, G. S., Medoff, B. D., Manice, L. A., Colvin, R. A., Luster, A. D. (2008). Development of a novel chemokine-mediated *in vivo* T cell recruitment assay. Journal of Immunological Methods*,* 331 *(*1–2*),* 127–139. 10.1016/j.jim.2007.12.002; 18206159 PMC2277098

[49] Martin-Fontecha, A., Thomsen, L. L., Brett, S., Gerard, C., Lipp, M. et al. (2004). Induced recruitment of NK cells to lymph nodes provides IFN-γ for T_H_1 priming. Nature Immunology*,* 5 *(*12*),* 1260–1265. 10.1038/ni1138; 15531883

[50] Hensbergen, P. J., Wijnands, P. G., Schreurs, M. W., Scheper, R. J., Willemze, R. et al. (2005). The CXCR3 targeting chemokine CXCL11 has potent antitumor activity *in vivo* involving attraction of CD8+ T lymphocytes but not inhibition of angiogenesis. Journal of Immunotherapy*,* 28 *(*4*),* 343–351. 10.1097/01.cji.0000165355.26795.27; 16000952

[51] Wenzel, J., Bekisch, B., Uerlich, M., Haller, O., Bieber, T. et al. (2005). Type I interferon-associated recruitment of cytotoxic lymphocytes: A common mechanism in regressive melanocytic lesions. American Journal of Clinical Pathology*,* 124 *(*1*),* 37–48. 10.1309/4EJ9KL7CGDENVVLE; 15923172

[52] Kozawa, K., Sekai, M., Ohba, K., Ito, S., Sako, H. et al. (2021). The CD44/COL17A1 pathway promotes the formation of multilayered, transformed epithelia. Current Biology*,* 31 *(*14*),* 3086–3097.e3087. 10.1016/j.cub.2021.04.078; 34087104

[53] Mao, F., Li, D., Xin, Z., D., Y., Wang, X. et al. (2020). High expression of COL17A1 predicts poor prognosis and promotes the tumor progression via NF-κB pathway in pancreatic adenocarcinoma. Journal of Oncology*,* 2020*,* 8868245. 10.1155/2020/8868245; 33381179 PMC7758145

[54] Wu, J., Li, Z., Zeng, K., Wu, K., Xu, D. et al. (2019). Key genes associated with pancreatic cancer and their association with outcomes: A bioinformatics analysis. Molecular Medicine Reports*,* 20 *(*2*),* 1343–1352. 10.3892/mmr.2019.10321; 31173193

[55] Thangavelu, P. U., Krenacs, T., Dray, E., Duijf, P. H. (2016). In epithelial cancers, aberrant COL17A1 promoter methylation predicts its misexpression and increased invasion. Clinical Epigenetics*,* 8 *(*1*),* 120. 10.1186/s13148-016-0290-6; 27891193 PMC5116176

[56] Yan, X., Zhang, C., Liang, T., Yang, F., Wang, H. et al. (2017). A PTEN-COL17A1 fusion gene and its novel regulatory role in Collagen XVII expression and GBM malignance. Oncotarget*,* 8 *(*49*),* 85794–85803. 10.18632/oncotarget.20526; 29156757 PMC5689647

[57] Iacobuzio-Donahue, C. A., Velculescu, V. E., Wolfgang, C. L., Hruban, R. H. (2012). Genetic basis of pancreas cancer development and progression: Insights from whole-exome and whole-genome sequencing. Clinical Cancer Research*,* 18 *(*16*),* 4257–4265. 10.1158/1078-0432.CCR-12-0315; 22896692 PMC3422771

