# Supplementary figures and images for: Prognostic-related genes for pancreatic cancer typing and immunotherapy response prediction based on single-cell sequencing data and bulk sequencing data
Source: Oncol Res. 2023 Jul 21;31(5):697–714. doi: 10.32604/or.2023.029458 (PMC10398398; doi:10.32604/or.2023.029458)

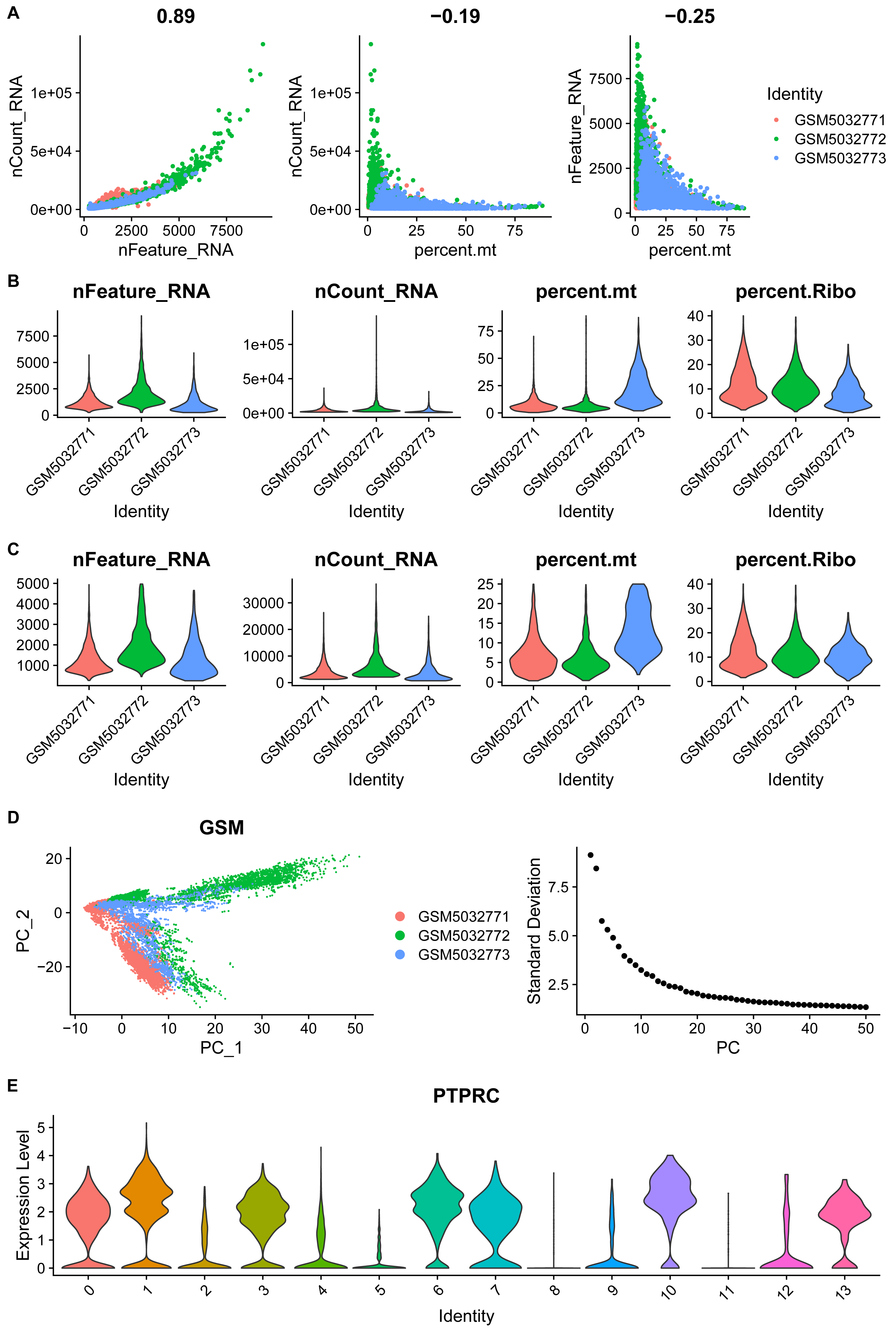

Supplement: FIGURE S1. [file OncolRes-31-29458-s001.tif]

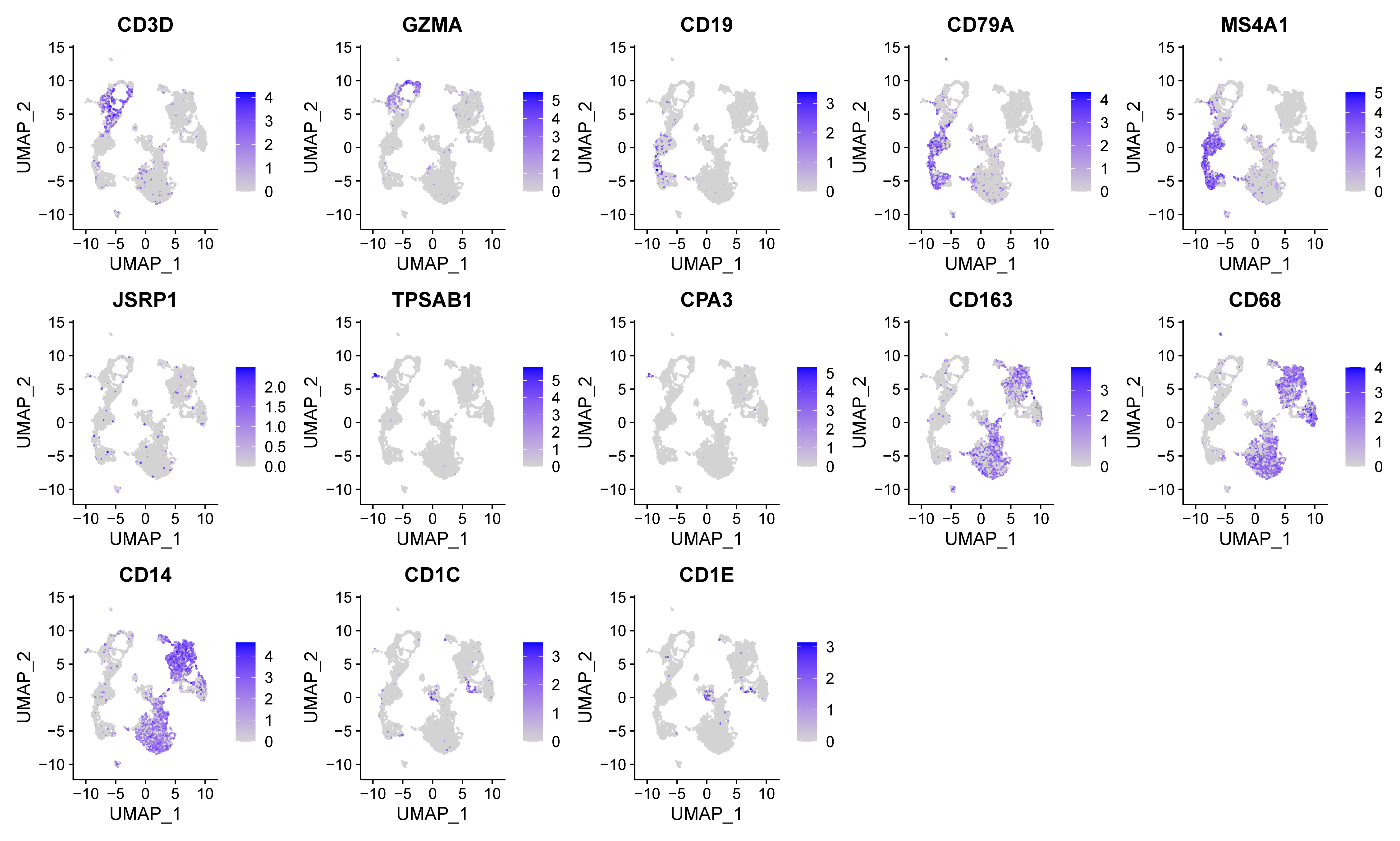

Supplement: FIGURE S2. [file OncolRes-31-29458-s002.tif]

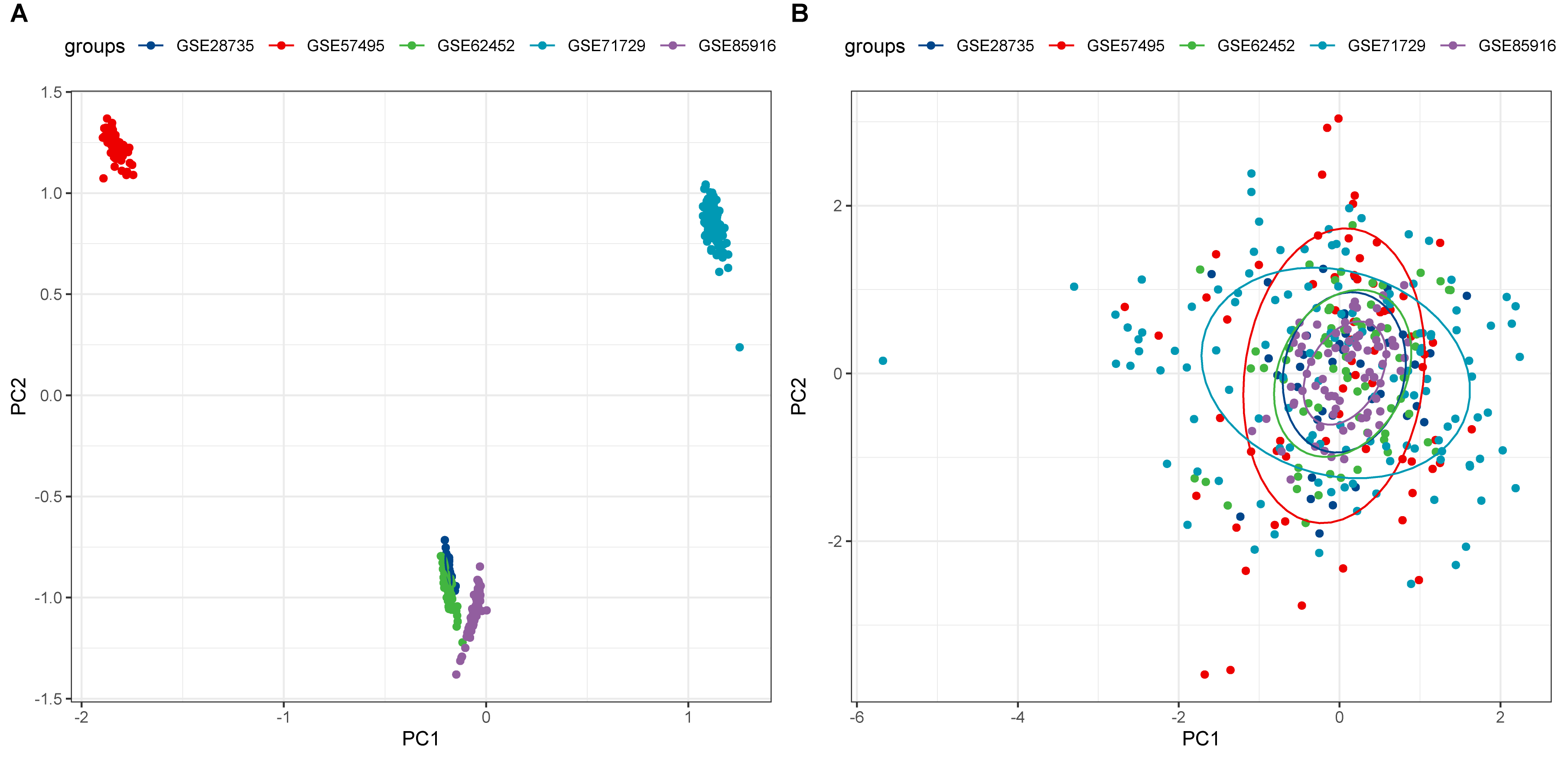

Supplement: FIGURE S3. [file OncolRes-31-29458-s003.tif]
